# Supplementary material for: Evaluation of the One Health-Ness of 20 Years of Antimicrobial Resistance Surveillance in Norway
Source: Antibiotics (Basel). 2023 Jun 21;12(7):1080. doi: 10.3390/antibiotics12071080 (PMC10376192; doi:10.3390/antibiotics12071080)
Supplement: Supplementary file 1 [file antibiotics-12-01080-s001.zip › antibiotics-2346997-supplementary.html]

OH-EpiCap report


# OH-EpiCap report

## OH-EpiCap Index and Dimensions

OH-EpiCap and Dimension indices represent mean scores over all questions, expressed as a percentage.

## Targets

This section shows the results of the twelve targets, divided across the three dimensions.  
  
 Scores range 1-4, with higher values suggesting better adherence to the One Health principle (better integration of sectors), and lower values suggesting improvements may be beneficial.  
  
 Users are encouraged to hover over data points to view a breakdown of each target score.

Targets demonstrating **good adherence** to One Health principles are: **Target 1.3 Resources, Target 2.4 Communication, Target 3.2 Collaborative added value, Target 3.3 Immediate and Intermediate outcomes, Target 3.4 Ultimate outcomes**.  
Targets that would **most benefit from improvement** are: **None**.

## Dimension 1: Organization

This section shows the results across all indicators within the four targets of Dimension 1 (Organization).  
  
 Scores range 1-4, with higher values suggesting better adherence to the One Health principle (better integration of sectors), and lower values suggesting improvements may be beneficial.  
  
 Indicators labelled in grey indicate a question was answered with NA. Users are encouraged to hover over plotted data points to view the wording of the chosen indicator level, and any comments that may have been added in connection with a particular question.  
  
 Indicators demonstrating **good adherence** to One Health principles are: **Common aim, Budget, Human resources, Adaptability to changes**.  
  
Indicators that would **most benefit from improvement** are: **Internal evaluation**.

## Dimension 2: Operations

This section shows the results across all indicators within the four targets of Dimension 2 (Operations).  
  
 Scores range 1-4, with higher values suggesting better adherence to the One Health principle (better integration of sectors), and lower values suggesting improvements may be beneficial.  
  
 Indicators labelled in grey indicate a question was answered with NA. Users are encouraged to hover over plotted data points to view the wording of the chosen indicator level, and any comments that may have been added in connection with a particular question.  
  
 Indicators demonstrating **good adherence** to One Health principles are: **Emergence**.  
  
Indicators that would **most benefit from improvement** are: **None**.

## Dimension 3: Impact

This section shows the results across all indicators within the four targets of Dimension 3 (Impact).  
  
 Scores range 1-4, with higher values suggesting better adherence to the One Health principle (better integration of sectors), and lower values suggesting improvements may be beneficial.  
  
 Indicators labelled in grey indicate a question was answered with NA. Users are encouraged to hover over plotted data points to view the wording of the chosen indicator level, and any comments that may have been added in connection with a particular question.  
  
 Indicators demonstrating **good adherence** to One Health principles are: **Improved knowledge, OH team, Strategy, Preparedness, Interventions, Advocacy, Awareness, Research, Policy changes**.  
  
Indicators that would **most benefit from improvement** are: **Operational cost**.
